# Large Scale Near-Duplicate Celebrity Web Images Retrieval Using Visual and Textual Features

**DOI:** 10.1155/2013/795408

**Published:** 2013-09-14

**Authors:** Fengcai Qiao, Cheng Wang, Xin Zhang, Hui Wang

**Affiliations:** College of Information Systems and Management, National University of Defense Technology, Changsha 410073, China

## Abstract

Near-duplicate image retrieval is a classical research problem in computer vision toward many applications such as image annotation and content-based image retrieval. On the web, near-duplication is more prevalent in queries for celebrities and historical figures which are of particular interest to the end users. Existing methods such as bag-of-visual-words (BoVW) solve this problem mainly by exploiting purely visual features. To overcome this limitation, this paper proposes a novel text-based data-driven reranking framework, which utilizes textual features and is combined with state-of-art BoVW schemes. Under this framework, the input of the retrieval procedure is still only a query image. To verify the proposed approach, a dataset of 2 million images of 1089 different celebrities together with their accompanying texts is constructed. In addition, we comprehensively analyze the different categories of near duplication observed in our constructed dataset. Experimental results on this dataset show that the proposed framework can achieve higher mean average precision (mAP) with an improvement of 21% on average in comparison with the approaches based only on visual features, while does not notably prolong the retrieval time.

## 1. Introduction

**Figure 1:**
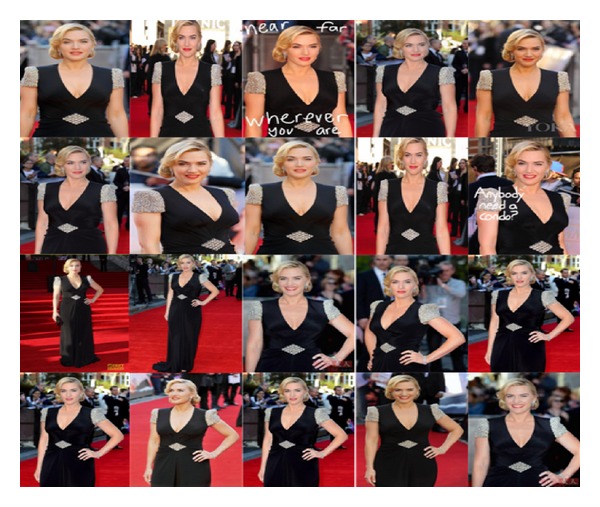
Examples of near-duplicate images contained in the top 1000 thumbnails returned by Google image search engine for the query “Kate Winslet” on January 8, 2013.

Near-duplicate images are defined as images of the same object or scene viewed under different imaging conditions [1]. Retrieval of near-duplicate images has boosted many real-world applications such as image annotation, image understanding [2], and content-based image retrieval (CBIR) [3]. It can improve traditional text-based image search by filtering duplicate query results, can be used as a bridge to relate two web pages in different languages, and can also provide similarity clues for recognizing visual events and searching news video clips. Among the tremendous amount of web images, celebrity images including posters, portraits, news photos, movie snapshots are of particular interest to the end users. Moreover, in agreement with the observation of [4], we find that near-duplication is also particularly prevalent in queries for celebrities and historical figures. For example, on January 8, 2013, the top 1000 thumbnails returned by the Google image search for the query “Kate Winslet” contained 51 images (see Figure 1 for some of them) about Kate's arriving in London for Titanic 3D premiere. These images are exactly the same with each other or slightly different in scales, cropping, and text insertion manners or captured in the same (3D) scene but with different cameras. 

Most recent near-duplicate image retrieval systems [5–9] adopt bag-of-visual-words (BoVW) model, which is a popular and practical method for large-scale image datasets. A typical BoVW based method includes routines which builds visual vocabulary, performs SIFT descriptors [10] quantization and online near-duplicate image retrieval. Because of the application of textual indexing and retrieval schemes, large scale near-duplicate retrieval becomes simple and effective. Nevertheless, the deficiency of current BoVW methods lies in the approximation error incurred by quantization and the loss of geometric relations between local features caused by the histogram-based representation adopted. Both factors reduce the discriminative power of local image features. Although many improvements such as hamming embedding (HE), weak geometry consistency (WGC), and enhanced weak geometry consistency (E-WGC) have been presented [1, 6, 11] to overcome these problems to some extent, they all depend solely on visual clues. In addition, to our best knowledge, no near-duplicate image retrieval methods have been experimented totally on celebrity image datasets. However, as well known, nearly all web images are surrounded with texts in their hosting webpage. By properly utilizing these surrounding texts we may get considerable improvements in the precision and recall of near-duplicate retrieval results. Hence, we propose in this paper a new text based data-driven reranking framework, which utilizes textual features and is combined with state-of-art BoVW schemes. Our main contributions to large scale near-duplicate celebrity web image retrieval are three pronged.

First, we construct a 2-million-celebrity-image-text dataset from the Internet about 1089 celebrities and divide near-duplicate celebrity web images into three categories in our ground-truth dataset according to our observation. All the images' surrounding texts are also kept and properly organized for further use. The necessities are listed below. (i) No existing available dataset provides images together with their accompanying text. (ii) None of them has focused only on celebrities or persons. (iii) Our constructed dataset provides fine-grain ground-truth labels, that is, the specific category of near-duplication of each near-duplicate image.

Second, we thoroughly evaluate six representative existing local visual feature based methods based on our constructed dataset. According to our experiments, BoVW with weak geometry consistency outperforms other methods when utilizing only visual feature.

Finally, to leverage the useful cues embedded in the surrounding texts of web images and hence to improve the precision and recall, we propose a text-based data-driven reranking framework. The framework improves the state-of-art local visual feature based BoVW methods through further exploiting the accompanying texts of images. It is worthwhile to note that the input of our framework is the same as BoVW schemes which means that we do not need to input the query image's accompanying text although we utilize text information in the retrieval process. Experiments on our constructed dataset of 2 million celebrity web images show that the proposed method achieves 21% improvement on average compared with pure BoVW methods.

The rest of this paper is organized as follows.  Section 2 discusses the related work. The celebrity web image-text dataset is introduced in Section 3, followed by our proposed data-driven reranking retrieval framework in Section 4. Experiments are presented and analyzed in Section 5. Finally, we conclude the paper in Section 6.

## 2. Related Work

Given a query image, near-duplicate image retrieval aims to find all images that are identified as essentially the same by human subjects. Near-duplicate image retrieval has been intensively addressed and researched in the last decade, partly attributed to the emergence of TRECVID benchmark for multimedia search. Researchers in this community often conduct their work in different application perspectives, typically including copyright protection, CBIR [12], and lately object or pattern recognition [13]. Generally, existing near-duplicate image retrieval methods make use of some global or local statistics of low-level features extracted from the query and database images. They often concatenate the (statistics of local) features into a single vector or matrix and use it as the image representation for similarity evaluation. Hence, we can largely categorize these methods into two groups, that is, the global feature based and the local feature based ones, which are overviewed separately as follows.

Global feature based algorithms have been researched for a long time. Wang et al. [14] proposed a fast and effective method in which each image is compactly represented by a K-bit hash code to detect all visually duplicate groups in large image collections. References [15, 16] extended the idea of Wang et al. [14] by adding the canny edge information to the hash code with the purpose of further improving the discriminative power of the image representation. 

In local feature based methods, bag-of-visual word (BoVW) scheme has been widely adopted in different contexts such as visual object recognition/classification, CBIR, and semantic concept detection. All these researches adopt BoVW as an image representation which involves routines for visual vocabulary construction, vector quantization, and pair-wise descriptor matching. However, it is notable that BoVW often produces many false matches which are mainly introduced by the vector quantization error. To tackle this issue, geometric verifications, for example, Hamming embedding (HE) [1] and weak geometric constraint (WGC) [1], have been proposed to prune these false visual word matches. Different from BoVW, Zhong et al. [9] presented a novel scheme where image features are bundled into local groups. Each group of bundled features becomes much more discriminative than a single local feature, and within each group simple and robust geometric constraints can be efficiently enforced. They achieved a 77% precision improvement over the baseline BoVW based approach and a 24% improvement over full geometric verification alone.

Before closing this section, we would emphasize that our research in near-duplicate celebrity web images retrieval adopts a methodology similar to the search-based images annotation [15, 17–19] rather than the traditional face recognition based approaches. In the emerging area of *Internet Vision* [20], the search-based scheme has been adopted by many researchers. Besides, the textual keyword based approaches have been successful applied to image search, while there are, to the best of our knowledge, very few reported researches that have applied textual information to near-duplicate images retrieval. In this paper we proposed a novel framework which combines BoVW schemes with textual information with the purpose of improving duplicate celebrity web image retrieval.

## 3. The Celebrity Web Image-Text Dataset

### 3.1. Data Collection

To collect celebrity web images, from January 8, 2013, to May 3, 2013, we constructed a celebrity dictionary using the 1089 top searched celebrity names in 5 areas. We use these names as query keywords and performed image search using Google image search engine and six news search engines including Google news, Yahoo news, Ifeng news, Sina news, Panguso news, and Baidu news. The URL patterns for each page of the returned images are listed in Table 1. For Google image search engine, we downloaded the returned 1000 images (at best) and the main text (including title, content, and image captions) on their hosted web pages for each query name. Images with exactly the same URLs are removed. For each news search engine, we crawled the latest 5 pages of returned results for each query, saving the news's text and accompanied images. Similarly, news web pages are also saved without the same URLs. The crawling and collection process lasted for nearly four months. In the end, we get more than 2 million images and their related text totally (see Table 2 for details). These data are organized in unit of image-text pairs which means that each image is related to some text. Each image-text pair is assigned with a unique ID to index the image and its related text.

### 3.2. Types of Near-Duplication and Ground-Truth Labeling

**Figure 2:**
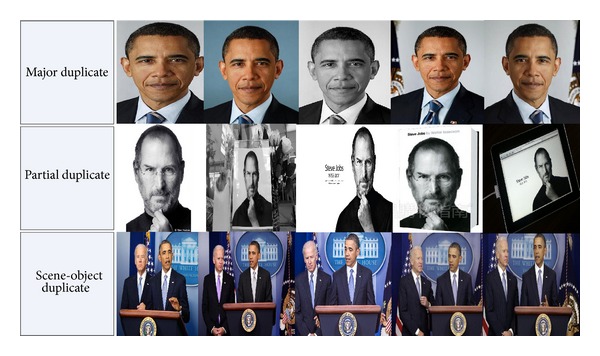
Examples of three categories of near-duplicate celebrity images.

We inspect a substantial number of these celebrity images to identify instances and types of near-duplication. According to our observation, three main categories of near-duplicates exist in these celebrity images (see Figure 2 for some examples of each category). (i) *Major duplicates* refer to images being exactly the same or images which are identified exactly the same by human subjects but have (slightly) different scales, colors, file formats, luminance intensities, and so forth. (ii) *Partial duplicates* refer to images of which some parts are exactly or fully duplicate to (some parts of) others. (iii) *Scene-object duplicates* are images sharing the same 3D scene or the same object (with object-class variability like gesture variation) but captured by different cameras at different time. Images in each ground-truth group include one or more near-duplicate categories. The geometric and photometric transformations related to each category of near-duplicates are detailed in Table 3. 

For the ground-truth labeling, we randomly select three celebrities from each area. Hence, 15 persons are selected in all. For each of them, we manually identify from his/her pictures collected the near-duplicate ones and their specific near-duplication types. In this way, we obtain the ground-truth composed of 57 groups of 2180 identified near-duplicate images. Each group contains images of all three categories of near-duplicates. The basic dataset contains near-duplicate images of our ground-truth images. As in [9], for evaluation purposes we identify and remove these duplicate images from the basic dataset by querying the database using every image from the ground-truth dataset and manually examining the returned images sharing any common parts with the query images. The number of ground-truth images in each type is listed in Table 4.

As listed in Table 4, major duplicates, partial duplicates, and scene-object duplicates account for almost the same in the ground-truth dataset, with 31.74%, 30.18%, and 38.08%, respectively. Many major duplicates exist because many websites just copy and publicize the original images without large modifications. Partial duplicates also occupy considerable large portion. It is noticeable that scene-object duplicates are very common for celebrity dataset. These images are usually taken on the same day and in the same event and hence often look like a series of snapshots of a period of video. From the subjective point of view, they also should be near-duplicate images.

## 4. Data-Driven Reranking Retrieval Framework

### 4.1. Framework

Near-duplicate celebrity web images retrieval is a process whose goal is to locate all major duplicate, partial-duplicate or scene-object images in a large corpus of web images given a query image *I*_*q*_:

(1) Iq→near-duplicate  images  retrieval{Ii ∣ Ij∈D,δ(Iq,Ij)=0,j=1,2,…,n}.

  
*D* is the basic dataset. *n* is the number of near-duplicate images retrieved. *δ*(*I*_*q*_, *I*_*j*_) is the near-duplicate estimation function which is defined as

(2) $$\begin{array}{c} \delta \left(I_{q},I_{j}\right)=\{\begin{array}{cc} 0, & \text{Sim}\left(I_{q},I_{j}\right)\geq \theta ,\\ 1, & \text{otherwise}. \end{array} \end{array}$$

Sim(*I*_*q*_, *I*_*j*_) is the similarity of *I*_*q*_ and *I*_*j*_. *θ* is the selected threshold got from heuristic manner or learning. The key of Sim(*I*_*q*_, *I*_*j*_) lies in image representation method and distance calculation. In the BoVW based near-duplicate image retrieval methods, an image is represented as a vector which is based on local features. Distances are calculated using cosine similarity and can be efficiently conducted by exploiting the inverted file index [21] (Section 4.2).

Denote the text context of top *k* images closest to query image *I*_*q*_ retrieved by BoVW based methods to be *Υ*_*q*_. Intuitively, *Υ*_*q*_ is most likely to contain related text information about the people appearing in the query image *I*_*q*_. From our observation, a celebrity's name, which affirmatively appears at least once in webpage title, content, or celebrity image captions, is a metric of vital importance to the celebrity images. Based on this observation, a two-stage solution for data-driven reranking retrieval framework is proposed:

- *BoVW-based search*, retrieving near-duplicate images of *I*_*q*_.
- *text-based reranking*, mining candidate celebrity names from *Υ*_*q*_, computing confidence scores, and updating the similarity of each *I*_*j*_.

**Figure 3:**
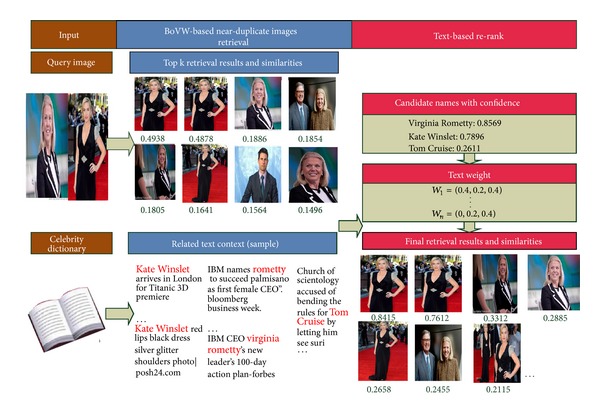
Framework for data-driven reranking retrieval.

Figure 3 illustrates the proposed data-driven reranking retrieval framework. Two major components in the framework are BoVW-based near-duplicate images retrieval and text-based reranking. The former step conducts near-duplicate images retrieval using BoVW-based methods which is only dependent on visual features. Then the later step reranks the retrieval results according to each image's text context feature. Finally, we get the retrieval results using both visual and text features.

### 4.2. BoVW-Based Near-Duplicate Images Retrieval

In this stage, we conduct near-duplicate images retrieval using BoVW methods. All the query images and images in the basic dataset are preprocessed firstly. SIFT descriptors are extracted from the images and clustering is carried out to quantize the descriptors into a visual vocabulary. Each descriptor in the image is then mapped to the nearest visual word in the vocabulary (vector quantization) and this forms a bag of visual words for each image. As the vocabulary is very large in this paper (1 M), to efficiently quantize without exhaustive search of the nearest visual words, we adopt a three-level vocabulary [1].

A bag of visual words is represented in the form of histogram, each bin of which accumulates the number of visual words found in the image. In practice, each bin of the histogram has its weight deriving from tf-idf (term frequency-inverse document frequency) method. Denote the histogram of an image *I*_*j*_ to be

(3) $$\begin{array}{c} I_{j}={\left(t_{1},\ldots ,t_{i},\ldots t_{m}\right)}^{T}. \end{array}$$

In (3), *m* is the vocabulary size and

(4) $$\begin{array}{c} t_{i}=\text{tf}\left(i\right)\times \text{idf}\left(i\right)=\frac{n_{ij}}{n_{j}}\times \ln\frac{N}{1+N_{i}}. \end{array}$$

In (4), *n*_*ij*_ is the number of the *i*th visual word appearing in *I*_*j*_. *n*_*j*_ is the total number of visual words in *I*_*j*_. *N* is the number of images in the basic dataset. *N*_*i*_ is the number of images in which the *i*th visual word appears. Denote *t*_*i*_(*I*_*q*_) and *t*_*i*_(*I*_*j*_) to be the weight of *i*th bin in image *I*_*q*_ and *I*_*j*_, respectively. The closeness between *I*_*q*_ and *I*_*j*_ is calculated via cosine similarity:

(5) $$\begin{array}{c} \text{Sim}\left(I_{q},I_{j}\right)=\frac{\sum_{i=1}^{m}t_{i}\left(I_{q}\right)\times t_{i}\left(I_{j}\right)}{\sqrt{\sum_{i=1}^{m}t_{i}{\left(I_{q}\right)}^{2}\sum_{i=1}^{m}t_{i}{\left(I_{j}\right)}^{2}}}. \end{array}$$

Sim(*I*_*q*_, *I*_*j*_) can be efficiently computed using inverted file indexing because the size of vocabulary is usually very large, and therefore the histogram is normally very sparse. In the inverted file indexing, each row stores the relationship of visual word and image. Local features like *x*, *y*, scale, orientation, and hamming code can be also stored in index cells dependent on the BoVW method used. Therefore, cosine similarity is evaluated only on a subset of images in the basic dataset (nonzero entries in the images).

In the retrieval process, to further speed up the online retrieval time we use hamming embedding (HE) [1] scheme. HE maintains a binary signature for each SIFT descriptor. The binary signature is stored in the inverted file index cells. During retrieval, any two matched visual words can be pruned if the hamming distance between their signatures is large than a threshold. At the same time, weak geometry consistency (WGC) [1] or enhanced weak geometry consistency (E-WGC) [11] can also be conducted and embedded into the inverted file indexing to fast reranking the images according to their geometric consistency with the query image. We name the BoVW method without HE and geometric consistency checking as traditional BoVW. As a result, there are six BoVW variations in all traditional BoVW, BoVW + HE, BoVW + HE + WGC, BoVW + HE + E-WGC, BoVW + WGC, and BoVW + E-WGC. 

### 4.3. Text-Based Reranking

As mentioned above, celebrity's name plays a vital role in the retrieval of near-duplicate celebrity images. Our text-based reranking scheme is dependent on the identification of celebrity names from the text context of the top *k* images retrieved by BoVW method. The top *k* images are most likely to contain the same person(s) appearing in the query image. Then, with the identified name or names, text retrieval can be efficiently performed using inverted file indexing. For images with text containing identified names, intuitively, the similarities of which should be larger than original values to some extent. In other words, there exist many false positive images whose similarities are bigger than some true positives when retrieval is conducted under large scale celebrity images dataset. By properly importing celebrity's name, we can filter many false positives which impossibly share any common parts with the query image, thus reranking true positive images to anterior order. 

#### 4.3.1. Candidate Name Identification and Confidence Computing

Firstly, we mine candidate names from *Υ*_*q*_ and compute each candidate name's confidence score. The method used in this step is mainly inspired by [15], but we adopt the idea with a very different purpose, that is, to discover the possible celebrities contained in the given image. Given the name dictionary, all the possible names are defined as

(6) $$\begin{array}{c} V=\left\{v_{l}\;|\;l=1,\ldots ,K\right\}, \end{array}$$

where *K* is the number of celebrities in our name dictionary. With the name dictionary and text context *Υ*_*q*_of the query image, one or more candidate names can be identified. Denote candidate names extracted from *Υ*_*q*_ to be *V*_*q*_ (*V*_*q*_ ⊂ *V*). Given a name *v*_*l*_^(*q*)^ ∈ *V*_*q*_, denote *p*(*v*_*l*_^(*q*)^ | *Υ*_*q*_) to be the confidence score of *v*_*l*_^(*q*)^. Denote the text features for *v*_*l*_^(*q*)^to be *T*(*v*_*l*_^(*q*)^). *T*(*v*_*l*_^(*q*)^) considers the following three aspects.

- *Type of names:* We consider a term in context *Υ*_*q*_ as a candidate name in two ways: (i) full name, for example, “Steve Jobs;” (ii) partial name, for example, “Steve” or “Jobs;” we observed that full name match was a very reliable indicator for the occurrences of celebrity names. Partial match can increase the confidence score because people often use partial names for simplicity.
- *Type of text:* in our basic dataset, three types of text related to a celebrity image are extracted and stored, that is, caption, page title, and content. Different types of text have different importance. For instance, information in page title or caption is more predictive of the identity of the celebrity than information in content.
- *Frequency:* frequency *f* corresponds to the number of times that *v*_*l*_^(*q*)^ occurs in *Υ*_*q*_. Intuitively, *v*_*l*_^(*q*)^ is more likely to appear in the query image if its frequency is high. In our experiment, *v*_*l*_^(*q*)^ is under consideration only if *f* ≥ 2.

The confidence score of *v*_*l*_^(*q*)^ is estimated by

(7) $$\begin{array}{c} p\left(v_{l}^{\left(q\right)}\;|\;\Upsilon_{q}\right)=\frac{1}{1+e^{-(W^{T}T(v_{l}^{\left(q\right)})+b)}}, \end{array}$$

where parameters *W* and *b* are learnt by logistic regression.

#### 4.3.2. Related Text Voting and Updating the Similarities

**Figure 4:**
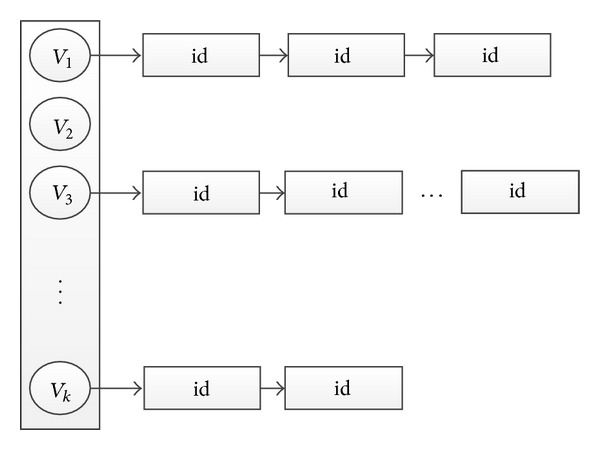
Structure of inverted file indexing used in related text retrieval.

In this step we first perform text retrieval using the names identified in (1) as query keywords to find text containing specified name. This procedure can be efficiently conducted using inverted file indexing, whose structure is illustrated in Figure 4. As each text-image pair has been assigned a unique ID in our basic dataset (Section 3.1), the index stores the name-ID relationship. There are *K* rows in all, each of which corresponds to a celebrity name and ids to the list of texts which contain the name. As a consequence, given a celebrity name, the texts are hashed into the index and the IDs of text containing this name are acquired immediately.

We divide the text of an image into three types: page title, content, and image caption. As each type has different importance to identify the celebrity image, the text information of image *I*_*j*_ can be represented by a vector

(8) $$\begin{array}{c} T_{j}=\left(W_{\text{title}},W_{\text{content}},W_{\text{caption}}\right). \end{array}$$

We assign the weights of *W*_title_, *W*_content_, and *W*_caption_ with 0.4, 0.2, and 0.4, respectively, according to experiential observation. To make clear, if the queried celebrity name appears in the title, then we assign *W*_title_ with value 0.4, otherwise 0. Same procedure is applied to content (title) and caption. As a result, we construct three inverted file indexing structures: page title index, content index, and caption index. For each query name *v*_*l*_^(*q*)^ ∈ *V*_*q*_, fast retrieval is conducted in the three indexing structures at the same time. Denote *S*(*v*_*l*_^(*q*)^) to be the retrieved text set for *v*_*l*_^(*q*)^:

(9) $$\begin{array}{c} S\left(v_{l}^{\left(q\right)}\right)=\left\{T_{j}\;|\;T_{j}\subset T,v_{l}^{\left(q\right)}\in T_{j}\right\}. \end{array}$$

Each element *T*_*j*_ in *S*(*v*_*l*_^(*q*)^) represents the text information of image *I*_*j*_. Denote *w*_*j*_ to be the score of text information *T*_*j*_. *w*_*j*_ is simply estimated by

(10) $$\begin{array}{c} w_{j}=W_{\text{title}}+W_{\text{content}}+W_{\text{caption}}. \end{array}$$

Finally, for each image *I*_*j*_ whose related texts are in *S*(*v*_*l*_^(*q*)^), we update its similarity to the query image according to both visual features and text features. The data-driven reranking framework adjusts the similarity computed in (5) by

(11) $$\begin{array}{c} \text{Si}{\text{m}}_{\text{final}}\left(I_{q},I_{j}\right)=2^{\gamma (p+w_{j})}\times \text{Sim}\left(I_{q},I_{j}\right). \end{array}$$

*p* is the confidence score of *v*_*l*_^(*q*)^ estimated by (7). *w*_*j*_ is the score of text information *T*_*j*_. The factor *γ* is a parameter used to control the influence of text. Thus, The original visual-only-based similarity Sim(*I*_*q*_, *I*_*j*_) is boosted, by a factor corresponding to the text information of image, for images whose related text information is in *S*(*v*_*l*_^(*q*)^) and *v*_*l*_^(*q*)^ ∈ *V*_*q*_. For images whose text does not contain any name in *V*_*q*_, the similarities of which stay unchanged.

## 5. Experimental Results

As pointed out in [1], near-duplicate is a kind of relationship for image pairs. Thus two natural measures are the precision and recall of duplicate image pairs. A “correct” image pair means it belongs to the intersection of a detected group and a ground-truth group. Then, the image pair precision (IPP) and image pair recall (IPR) are defined, respectively, as

(12) $$\begin{array}{c} \text{IPP}=\frac{\#\text{correct}\;\text{pairs}}{\#\text{detected}\;\text{pairs}}*100\%,\\ \text{IPR}=\frac{\#\text{correct}\;\text{pairs}}{\#\text{groud}-\text{truth}\;\text{pairs}}*100\%. \end{array}$$

As mentioned in Section 3.2, the ground-truth dataset is composed of 57 groups of 2180 identified near-duplicate images. In our evaluation, we randomly select two images from each ground-truth group as query images and hence obtain 114 queries in all. Following [9] we use mean average precision (mAP) as our evaluation metric. For each query image we do the retrieval work and compute its precision-recall curve, from which we obtain its average precision and then take the mean value over all queries as the mAP. To evaluate the performance with respect to the size of the dataset, we also build four smaller datasets (50 K, 500 K, 1000 K, and 1500 K) by sampling the basic dataset.

### 5.1. Evaluation

#### 5.1.1. Baseline Methods

As mentioned in Section 4.2,we use six BoVW based methods in all. They are traditional BoVW, BoVW + HE, BoVW + HE + WGC, BoVW + HE + E-WGC, BoVW + WGC, and BoVW + E-WGC, making up of our baseline methods. To ensure the coverage of vocabulary, we choose a visual vocabulary of 1 M visual words for the two-million-basic dataset.

#### 5.1.2. *mAP* Comparisons

Each of the six BoVW variations can be combined with our data-driven reranking framework. As a result, 12 methods are compared with each other. In our implementation, the hamming code in HE is 32 and the threshold for hamming distance is set at 15. The parameter *γ* in (11) is set to be 0.8.

**Figure 5:**
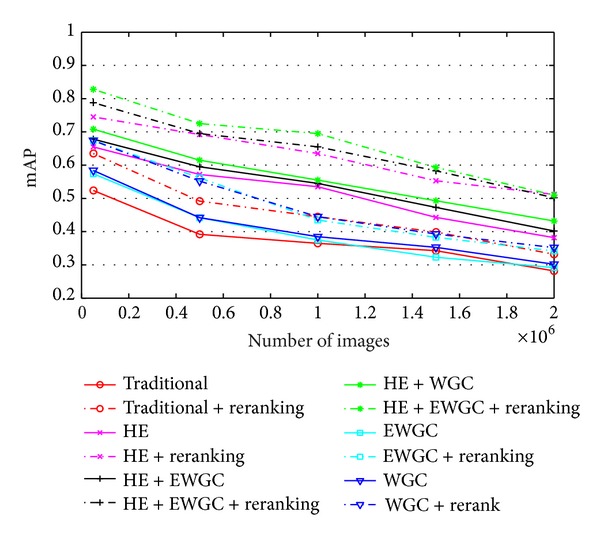
Comparison of different methods using mAP: the six BoVW methods without text-based reranking are in solid lines. Dashed lines are the BoVW methods combined with our data-driven reranking framework.

Figure 5 compares the above 12 methods using mAP, leading to three observations. First, our data-driven reranking framework boosts all the six BoVW methods which are dependent only on visual features. On the 2-million dataset, the improvements of mAP for traditional BoVW, BoVW + HE, BoVW + HE + WGC, BoVW + HE + E-WGC, BoVW + WGC, and BoVW + E-WGC are 17%, 34%, 25%, 18%, 16%, and 17%, respectively, 21% on average. Second, as shown by the curves labeled “HE + WGC” and “HE + WGC+ re-rank”, BoVW method outperforms other BoVW methods with and without our text-based reranking stage. Finally, the hamming embedding is of vital importance to the near-duplicate celebrity images retrieval. On the 2-million dataset, when combining HE with our reranking stage, BoVW + HE, BoVW + HE + WGC and BoVW + HE + E-WGC all achieve almost the same high mAP than others, with highest mAP 0.508 of BoVW + HE + WGC (79% improvement over the traditional BoVW without HE and our reranking).

#### 5.1.3. Impact of Factor *γ*

The factor *γ* in (11) is a parameter used to control the influence weight of text information. We test the performance of our data-driven reranking framework using different *γ* values on the 2-million dataset. As Table 5 shows, the mAP achieves highest value when *γ* is 0.8. 

#### 5.1.4. Runtime

We perform our experiments on a personal computer equipped with a single Core (TM) i5-2320 3.0 GHz CPU and 16 Gigabytes of RAM.  Table 6 shows the average query time of BoVW + HE + WGC method and BoVW + HE + WGC plus our text-based reranking on the 2-million dataset. As can be seen, our reranking stage only introduces a modest time penalty (0.8 seconds). That is because we use inverted file indexing for fast text retrieval. 

#### 5.1.5. mAP of Each Near-Duplicate Category

**Figure 6:**
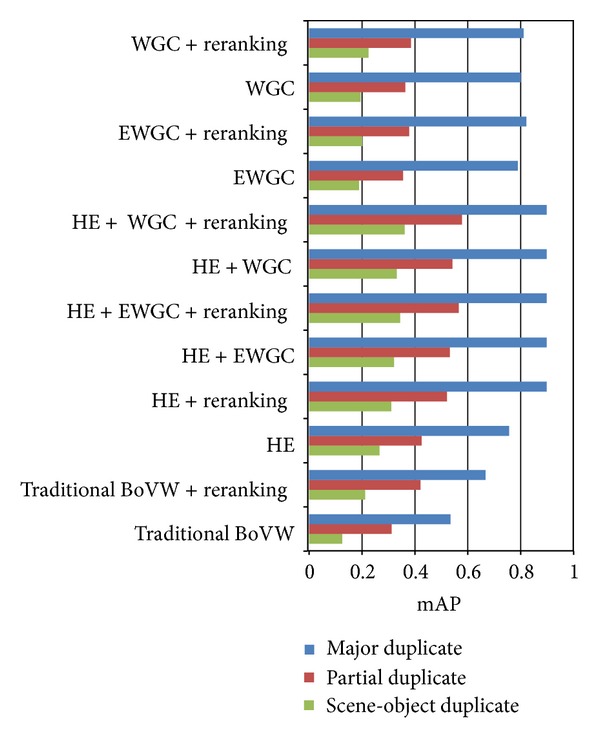
mAP with respect to each near-duplicate category on the 2-million dataset.

In our ground-truth, each group contains images of all three categories of near duplicates. As a result, we also compute the mAP with respect to each near duplicate category with the goal of further investigating and comparing the capability of our method to identify different categories of near duplicates. We conduct the comparison on the 2-million dataset. As Figure 6 shows, major duplicate is easy to identify for all methods, while scene-object duplicate is hardest and partial duplicate in the middle. The mAPs of all categories are improved after our data-driven reranking stage. Obviously, “HE + WGC + re-rank” outperforms all the other methods especially for scene-object duplicate and partial duplicate.

### 5.2. Sample Results

**Figure 7:**
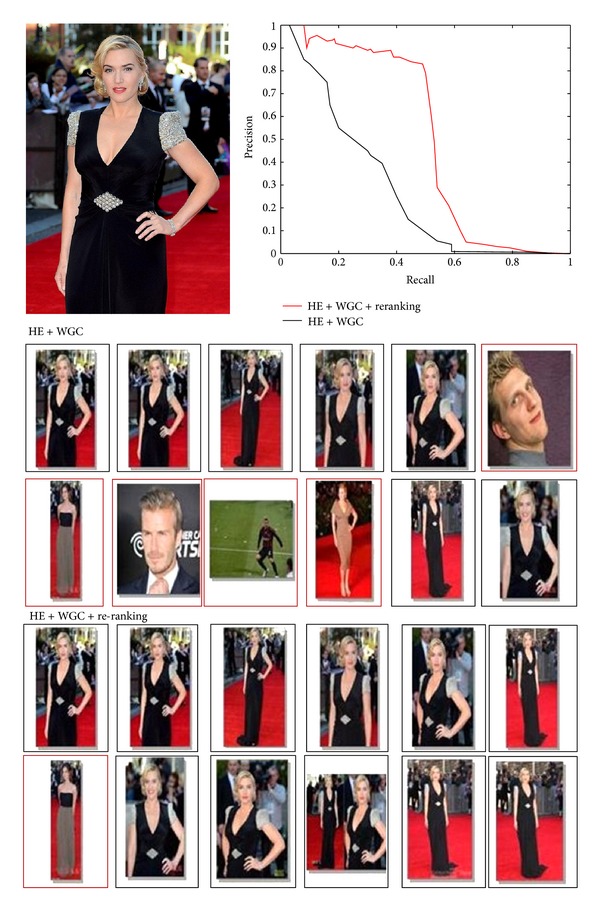
Top 12 images returned by BoVW with HE and WGC and BoVW with HE and WGC plus our text-based reranking. False positives are marked by red rectangles.

Figure 7 gives example for our results on the 2-million dataset. We show the top 12 images returned by BoVW with HE and WGC and BoVW with HE and WGC plus our text based reranking. False positives are marked by red rectangles. The image at top left is the query image and top-right the precision-recall curves for the above two methods. It is obvious that our text-based reranking method filters many false positive images such as Beckham though they contain many visual local patches similar to those in the query image. For this query, we improve the mAP from 0.321 to 0.52, a 62% improvement.

## 6. Conclusions

We have proposed a text-based data-driven reranking framework. The framework is combined with six state-of-art BoVW schemes and improves the near-duplicate celebrity images retrieval results a lot. We also construct a 2-million-celebrity-image-text dataset with 1089 celebrities' name query and divide near-duplicate celebrity web images into three categories in our ground-truth dataset according to our observation. 

In the future work, we plan to improve our text-based reranking method by exploiting more text information not only celebrity names. After all, the text processing methods used are to some extent quite basic and nearly naïve. There is room for improvement in the future in this area. For example, finding events or topic from the text may also be more benefit for near-duplicate celebrity images retrieval. At the same time, we also want to attempt to research the use of text in general near-duplicate images retrieval not only celebrity images.

## Figures and Tables

**Table 1:** URLs of the search engines used in the experiments.

Search engine	URL pattern
Google image	http://www.google.com.hk/search?tbm=isch&amp;q=&lt;k&gt;&amp;start=&lt
Google news	http://www.google.com.hk/search?q=&lt;k&gt;&amp;tbm=nws&amp;start=&lt
Yahoo news	http://news.yahoo.cn/s?q=&lt;k&gt;&amp;page=&lt
Ifeng news	http://search.ifeng.com/sofeng/search.action?q=&lt;k&gt;&amp;p=&lt
Sina news	http://search.sina.com.cn/?q=&lt;k&gt;&amp;c=news&amp;page=&lt
Panguso news	http://news.panguso.com/newssearch.htm?q=&lt;k&gt;&amp;p=&lt
Baidu news	http://news.baidu.com/ns?tn=news&amp;rn=20&amp;word=&lt;k&gt;&amp;pn=&lt

**Table 2:** Number of the crawled celebrity images.

Category	# Names	# Images (Google)	# Images (news)	# Images (total)
Politicians	245	219030	171255	390285
Actors	312	423072	218398	641470
Singers	212	281112	211788	492900
IT leaders	189	142254	157657	299911
Sports stars	131	130519	114930	245449

Total	1089	1195987	874028	2070015

**Table 3:** The Image alterations related to each category of near-duplicates.

Near-duplicate category	Alterations
Major duplicate	Typical image alterations include adding logos or inserting texts, changes of pixel color vectors, color space, file format, or pixel luminance intensities, and some geometric transforms such as scaling, mirroring, and rotation.
Partial duplicate	Alterations undergone commonly include cropping, pasting an image to overlay a part of another, zooming, and adding border,
Scene-object duplicates	Sharing the same scene or undergone object-class variability. Variations between near duplicate images of this category are often caused by alterations such as viewpoint changes, partial occlusion, and differences in (intrinsic/extrinsic) camera parameters.

**Table 4:** Number of ground-truth images in each category.

Near-duplicate category	# Images	Percentage
Major duplicate	692	31.74%
Partial duplicate	658	30.18%
Scene-object duplicate	830	38.08%

Total	2180	100%

**Table 5:** Comparing the performance using different values of *γ*.

*γ*	0.2	0.4	0.6	0.8	1
mAP	0.488	0.493	0.501	0.508	0.499

**Table 6:** Average query time (not including feature extraction time).

BoVW + HE + WGC	BoVW + HE + WGC + re-ranking
2.9 s	3.7 s
